# Global DNA methylation levels are altered by modifiable clinical manipulations in assisted reproductive technologies

**DOI:** 10.1186/s13148-017-0318-6

**Published:** 2017-02-06

**Authors:** Jayashri Ghosh, Christos Coutifaris, Carmen Sapienza, Monica Mainigi

**Affiliations:** 1 Center for Research on Reproduction and Women’s Health, University of Pennsylvania School of Medicine, Philadelphia, PA 19104 USA; 2 Department of Obstetrics & Gynecology, University of Pennsylvania School of Medicine, 3701 Market Street, 8th Floor, Philadelphia, PA 19104 USA; 3 Fels Institute for Cancer Research and Molecular Biology, Temple University School of Medicine, Philadelphia, PA 19140 USA; 4 Department of Pathology and Laboratory Medicine, Temple University School of Medicine, Philadelphia, PA 19140 USA

**Keywords:** Methylation, Modifiable factors, IVF, Oxygen tension, Fresh transfer, Frozen transfer, *LINE1*

## Abstract

**Background:**

We analyzed placental DNA methylation levels at repeated sequences (*LINE1* elements) and all CCGG sites (the LUMA assay) to study the effect of modifiable clinical or laboratory procedures involved in in vitro fertilization. We included four potential modifiable factors: oxygen tension during embryo culture, fresh embryo transfer vs frozen embryo transfer, intracytoplasmic sperm injection (ICSI) vs conventional insemination or day 3 embryo transfer vs day 5 embryo transfer.

**Results:**

Global methylation levels differed between placentas from natural conceptions compared to placentas conceived by IVF. Placentas from embryos cultured at 20% oxygen showed significant differences in *LINE1* methylation compared to in vivo conceptions, while those from embryos cultured at 5% oxygen, did not have significant differences. In addition, placentas from fresh embryo transfer had significantly different *LINE1* methylation compared to placentas from in vivo conceptions, while embryos resulting from frozen embryos were not significantly different from controls. On sex-stratified analysis, only males had significant methylation differences at *LINE1* elements stratified for the modifiable factors. As expected, *LINE1* methylation was significantly different between males and females in the control population. However, we did not observe sex-specific differences in the IVF group. We validated this sex-specific observation in an additional cohort and in opposite sex IVF twins.

**Conclusion:**

We show that two clinically modifiable factors (embryo culture in 5 vs 20% oxygen tension and fresh vs frozen embryo transfer) are associated with global placental methylation differences. Interestingly, males appear more vulnerable to such treatment-related global changes in DNA methylation than do females.

**Electronic supplementary material:**

The online version of this article (doi:10.1186/s13148-017-0318-6) contains supplementary material, which is available to authorized users.

## Background

Assisted reproductive technologies (ART) have been associated with multiple epigenetic changes including alteration in DNA methylation in both the placenta and the offspring [1–7]. Several explanations have been proposed as contributing to the etiology of these changes, including infertility itself, superovulation, in vitro fertilization (IVF), gamete/embryo manipulation, and embryo culture [8, 9]. We have recently demonstrated, using an analysis of placentas from donor oocyte recipients, that site-specific DNA methylation differences observed between ART and in vivo conceptions are associated, at least in part, with the ART procedure itself, and not the underlying infertility [10]. Animal studies have confirmed that multiple techniques utilized during IVF may play a role in these methylation changes [11–19].

Collectively, these observations suggest strongly that some of the clinical or laboratory procedures used in ART are responsible for altering DNA methylation levels at multiple sites in the genome. Given this likelihood, we chose to compare modifications of several ART procedures, individually, for an association with DNA methylation differences. The clinical and laboratory procedures compared were embryo culture in 5 vs 20% O_2_, fresh embryo transfer vs frozen embryo transfer, intracytoplasmic sperm injection (ICSI) vs conventional insemination and day 3 embryo transfer vs day 5 embryo transfer. We used surrogate measures of genome-wide DNA methylation as biomarkers of whether exposure to individual clinical or laboratory protocols used in ART might alter epigenetic marks in the placenta. The rationale behind using measures of methylation level that are averaged over hundreds-of-thousands-to-millions of CpG sites rather than methylation levels at specific sites is that a surrogate genome-wide average is more likely to be representative of the degree to which a particular factor is capable of disrupting epigenetic marks. In addition, multi-site methylation differences that are cumulative and observed between groups stratified on the basis of particular exposures are more likely to be reproducible than differences at individual sites because the exposures being investigated are not hypothesized to have effects that are targeted at specific CpGs. Moreover, the collection of CpGs usually interrogated by site-specific, array-based profiling methods are highly-selected and enriched for gene promoters and gene bodies. Interrogating methylation levels at repeated sequences that make up a large fraction of the human genome or all sites recognized by a particular restriction endonuclease is more likely to be representative of the genome, as a whole, than even large numbers of CpG sites selected to be within or adjacent to coding sequences. Finally, searching for procedure-specific, site-specific methylation differences using array-based epigenome-wide profiling methods requires significant statistical penalties because of the requirement of correction for multiple testing.

The aim of this study was to analyze the placental DNA methylation levels at repeated sequences (*LINE1* elements) and all CCGG sites (the LUMA assay) to study the effect of modifiable clinical or laboratory procedures involved in in vitro fertilization. We included four potential modifiable factors: oxygen tension during embryo culture, fresh embryo transfer vs frozen embryo transfer, intracytoplasmic sperm injection (ICSI) vs conventional insemination or day 3 embryo transfer vs day 5 embryo transfer.

## Methods

### Samples and clinical protocols

The present study was approved by the University of Pennsylvania Institutional Review Board (approval number 804530). Placentas were acquired from live-born deliveries resulting from IVF pregnancies and naturally conceived pregnancies (controls). For IVF pregnancies, all patients had undergone in vitro fertilization at Penn Fertility Care (between 2006–2012 for original cohort and 2013 onwards for validation cohort) using standard protocols. Superovulation was performed using recombinant or purified-urinary follicle stimulating hormone and/or human menopausal gonadotropin. Gonadotropin dose was chosen based on patient characteristics and was adjusted during stimulation as clinically indicated based on patient response. Oocyte maturation was induced with human chorionic gonadotropin or leuprolide acetate followed by transvaginal egg retrieval 35–36 h later. Fertilization, by intracytoplasmic sperm injection (ICSI) was performed for either male factor or unexplained infertility as clinically indicated. Conventional insemination or ICSI and embryo culture were performed utilizing appropriate media (VitroLife; Gothenburg, Sweden) in microdroplets under oil in either 20% oxygen tension (5% CO_2_ in air) or 5% oxygen tension (5% O_2_, 5% CO_2_, and 90% N_2_), and transferred to the uterus on either day 3 (cleavage) or day 5 (blastocyst) of embryo development. Luteal support was provided by intramuscular progesterone (50 mg). Frozen embryos were cryopreserved at either the pronuclear or blastocyst stage using a slow-cooling protocol and subsequently thawed and transferred in a hormonally programmed cycle utilizing increasing doses of oral micronized estradiol (2–6 mg) followed by intramuscular progesterone (25–50 mg) to induce a receptive endometrium and to determine the timing of transfer.

### DNA preparation and bisulfite conversion

Placentas were collected at delivery and processed for DNA analysis within 5 h as previously described [1, 20]. Briefly, placental tissue (1.5–2.5 cm^3^) was excised from the fetal surface of the placenta, directly behind the cord insertion site. The sample was rinsed extensively with sterile saline solution to minimize maternal blood contamination. The tissue was transferred to a 15-ml Falcon tube for DNA extraction and initially stored at 4 °C; nucleic acid extractions were performed within 2–4 days of collection. Placenta genomic DNA was extracted using standard phenol-chloroform extraction methods. The isolated DNA was dissolved in TrisCl (10 mM, pH 8.0) and stored at −80 °C until further use. Unmethylated cytosine in genomic DNA (0.5–1 μg) was converted to uracil by treatment with sodium bisulfite using the EZ DNA Methylation KitTM (Zymo Research Corp., USA), following the manufacturer’s guidelines. The bisulfite-converted DNA was dissolved in 20-μl TrisCl (10 mM, pH 8.0) buffer and stored at −20 °C until further use.

### Luminometric methylation assay (LUMA)

Luminometric methylation assay (LUMA) was used to estimate global methylation levels of placental samples by sampling the fraction of the 2.3 million CCGG sites that are methylated. The protocol has been described previously [21]. We used a modification of this assay [22]. In short, placental genomic DNA (300 ng) was subjected to parallel digestions by methylation insensitive restriction endonuclease (*MspI*), and its methylation sensitive isoschizomer (*HpaII*) to produce 5′-CG overhangs. *EcoRI* was used in both the reactions for normalization, thus, resulting in 5′AATT overhangs. The digested samples were pyrosequenced (PyroMark Q96 ID, Qiagen) using the nucleotide dispensation order: GTGTCACATGTGTG. The peak heights corresponding to *HpaII* or *MspI* and *EcoRI* were then used to calculate the genomic DNA methylation fraction using the formula 1-[*HpaII*(G)/*EcoRI*(T)]/[*MspI*(G)/*EcoRI*(T)].

### *LINE1* methylation

Genomic DNA was treated with sodium bisulfite using the EZ DNA methylation kit (Zymo Research). Bisulfite converted DNA was amplified using primers and pyrosequenced as described previously [23]. *LINE1* methylation levels were estimated by taking the average of the first three CpGs.

### Statistical analysis

Statistical analysis was performed using GraphPad Prism (ver. 6.0 for Mac OS). Between-group differences in continuous data were analyzed using a two-tailed unpaired *t* test for two groups and one-way ANOVA for more than two groups. Tukey’s post hoc test was applied for multiple comparisons. Categorical data was analyzed using Fisher’s exact test for 2 × 2 contingency tables and a chi-square test for higher order contingency tables. Differences between males and females were analyzed using one-tailed unpaired *t* test due to prior expectation [24] for controls and singleton IVF; and one-tailed paired *t* test for opposite sex twins.

## Results

### Sample characteristics

Placental samples from 259 singleton pregnancies (182 ART- and 77 in vivo-conceived-controls) were analyzed by LUMA. High quality *LINE1* pyrosequencing assay data were also obtained for a subset of the 259 singleton pregnancies (126 ART and 65 control placental samples). Samples that did not pass pyrosequencing quality control (absent peaks or broad peaks) were not included for *LINE1* analysis. *LINE1* pyrosequencing was also done in the validation cohort (57 controls and 55 ART) and opposite sex twins (39 pairs). The demographic profile and relevant clinical characteristics of all samples are shown in Table 1.

**Table 1:** Demographic profile and clinical characteristics of the study subjects

	Original cohort			Validation cohort			Twin cohort
	ART (*n* = 182)	Controls (*n* = 77)	*P* value	ART (*n* = 55)	Controls (*n* = 57)	*P* value	ART (*n* = 39 pairs)
Maternal age (years, mean ± SD)	34.7 ± 3.6	32.2 ± 4.8	**<** *0.0001* ^a^	35.8 ± 3.9	29.5 ± 6.1	**<** *0.0001* ^a^	37.2 ± 5.2
Paternal age (years, mean ± SD)	36.9 ± 5.7	33.3 ± 5.2	**<** *0.0001* ^a^	38.3 ± 6.5	34.8 ± 4.6	0.0610	39.0 ± 6.9
Mode of egg fertilization (ICSI/non ICSI)	54/127	NA	–	30/23	NA	–	14/24
Oxygen tension (20/5%)	123/59	NA	–	16/38	NA	–	19/19
Type of embryo transfer (fresh/frozen)	128/54	NA	–	33/22	NA	–	26/13
Embryo transfer day (day 3/day 5)	110/72	NA	–	17/37	NA	–	18/20
Gestational age (weeks, mean ± SD)	39.2 ± 1.3	39.1 ± 1.2	0.4306^a^	38.2 ± 3.6	38.2 ± 6.4	0.9990^a^	36.5 ± 2.1
Birth weight (grams, mean ± SD)	3441.5 ± 498.6	3318.8 ± 559.3	0.0837^a^	3196 ± 918.4	3411 ± 541.7	0.1324^a^	2653.1 ± 576.9
Males (%)	83(45.6)	39 (50.6)	0.4972^b^	26	29	0.7103^b^	39(50.0)
Females (%)	99(54.4)	38 (49.4)		29	28		39(50.0)

*ART* assisted reproductive technology; *SD* standard deviation;

I*CSI* intracytoplasmic sperm injection; *NA* not applicable

^a^Unpaired two-tailed *t* test

^b^Fisher’s exact test

Values in italics denote significance

The control and ART groups differed significantly in both maternal and paternal age (*p* < 0.0001), with ART parents being 3–4 years older. Given the significant difference in parental age, we stratified each population to test for methylation differences associated with parental age (Additional file 1), based on a prior definition of “advanced parental age” (>35 vs ≤35 years; [25]). We tested four potentially modifiable clinical and laboratory factors used in ART for an effect on methylation level (Table 1 and Additional file 2). These modifiable factors were as follows: (1) mode of egg fertilization (ICSI or conventional insemination); (2) oxygen tension during embryo culture (20 or 5%); (3) type of embryo transfer (fresh or frozen); (4) embryo transfer day (day 3 or day 5).

### Surrogate measures of global methylation in ART vs controls

**Fig. 1:**
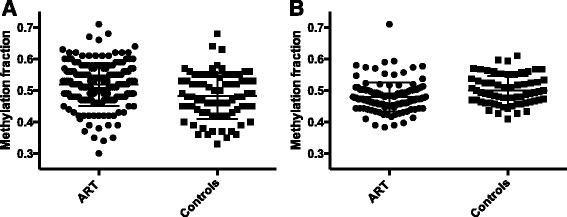
Average global methylation fraction of placental samples in the original cohort: methylation fraction of CCGG sites using LUMA assay (**a**); methylation fraction of *LINE-1* elements using pyrosequencing assay (**b**)

The LUMA assay measures methylation level averaged over 2.3 million CCGG sites (approximately 8% of the 28 million CpG sites in the human genome), and the *LINE1* assay measures average methylation levels at three CpG sites in 516,000 copies of *LINE1* elements (approximately 5.5% of the CpG sites in the genome). CCGG site methylation levels were significantly different (*p* = 0.0002) between controls and ART (Fig. 1a and Table 2) as were *LINE1* element methylation levels (*p* = 0.0034) (Fig. 1b and Table 3). Given these global differences between the control and ART groups, we stratified each group according to parental age and stratified the ART group by four clinical/laboratory procedures to determine whether differences were associated with one or more of these factors.

**Table 2:** Luminometric methylation assay (LUMA)

Criteria for comparison	Groups (*n*)	Global methylation fraction (mean ± SD)	*P* value	
			ANOVA	Tukey’s post hoc test (vs controls)
	Controls (77)	0.4827 ± 0.0723	–	–
Total ART	ART (182)	0.5182 ± 0.0670	–	*0.0002* ^a^
Mode of egg fertilization	ICSI (54)	0.5224 ± 0.0533	*0.0009*	**<** *0.01*
	Non ICSI (127)	0.5158 ± 0.0720		**<** *0.01*
Oxygen tension	20%(123)	0.5176 ± 0.0689	*0.0009*	**<** *0.01*
	5%(59)	0.5195 ± 0.0634		**<** *0.01*
Type of embryo transfer	Fresh (128)	0.5109 ± 0.0684	**<** *0.0001*	**<** *0.05*
	Frozen (54)	0.5357 ± 0.0608		**<** *0.0001*
Embryo transfer day	Day 3 (110)	0.5212 ± 0.0689	*0.0007*	**<** *0.001*
	Day 5 (72)	0.5138 ± 0.0643		**<** *0.05*

*ANOVA* analysis of variance; *ART* assisted reproductive technology; *SD* standard deviation; *ICSI* intracytoplasmic sperm injection

^a^Unpaired two-tailed *t* test

Values in italics denote significance

**Table 3:** *LINE1* methylation pyrosequencing assay

Criteria for comparison	Groups (*n*)	Global methylation fraction (mean ± SD)	*P* value	
			ANOVA	Tukey’s post hoc test (vs controls)
	Controls (65)	0.4997 ± 0.0463	–	–
Total ART	ART (126)	0.4789 ± 0.0459	–	*0.0034* ^a^
Mode of egg fertilization	ICSI (39)	0.4732 ± 0.0322	*0.0090*	**<** *0.05*
	Non ICSI (87)	0.4815 ± 0.0508		**<** *0.05*
Oxygen tension	20%(73)	0.4781 ± 0.0526	*0.0135*	**<** *0.05*
	5%(53)	0.4799 ± 0.0352		ns
Type of embryo transfer	Fresh (90)	0.4784 ± 0.0487	*0.0137*	**<** *0.05*
	Frozen (36)	0.4800 ± 0.0385		ns
Embryo transfer day	Day 3 (67)	0.4783 ± 0.0529	*0.0137*	**<** *0.05*
	Day 5 (59)	0.4796 ± 0.0463		**<** *0.05*

*ANOVA* analysis of variance; *ART* assisted reproductive technology; *SD* standard deviation; *ICSI* intracytoplasmic sperm injection; *ns* non significant

^a^Unpaired two-tailed *t* test

Values in italics denote significance

#### Note on assay reproducibility and directional difference between LUMA and *LINE1* methylation levels

Though the absolute differences observed in methylation between ART and control patients is small (Tables 2 and 3), the differences are highly statistically significant. In addition, multiple studies, particularly from the oncology literature, have shown a lack of correlation between methylation levels measured by LUMA and *LINE1* assays [26, 27]. This lack of correlation between the two assays is likely due to the fact that they interrogate different compartments of the epigenome (see Discussion).

In terms of the reproducibility of the techniques, many of the samples have been run multiple times. We have calculated the standard deviation and also used Bland–Altman plots to test for reproducibility. According to Bland–Altman [28], if 95% of the samples are within the limits of agreement then the assay is reproducible. In our data, 96.5% of samples are within the limits for the LUMA assay, and 100% of samples are in range for the *LINE1* assay.

### Parental age

We divided the samples on the basis of maternal and paternal age (> or ≤35 years of age; Additional file 1). The paternal age data were missing for some ART samples where anonymous sperm donors were used (*n* = 7). In the control group, paternal age data were missing for 14 samples. For both maternal and paternal age, global methylation differences between ART and controls, as measured by both LUMA and *LINE1*, were highly significant in the younger parental age group (≤35 years), suggesting that the difference in methylation between ART and control groups (Additional file 1) is not a result of the older average age of ART patients. We did not observe a significant difference in global methylation between ART and controls in the older parental age group though the number of controls in this comparison was small (LUMA: *n* = 22 for maternal age and *n* = 26 for paternal age; *LINE1*: *n* = 16 for maternal age and *n* = 21 for paternal age) (Additional file 1).

### Mode of egg fertilization

Nearly 30% of the ART samples resulted from eggs that were fertilized using intra cytoplasmic sperm injection (ICSI) (Table 1). We compared methylation of CCGG sites in the placentas between ICSI and conventional insemination subgroups but did not observe any significant differences (data not shown). On the other hand, each subgroup was significantly different from controls in methylation levels (*p* < 0.01). Overall, ANOVA indicated that the three groups showed a significant difference in methylation levels (*p* = 0.0009) (Table 2).

Similarly, *LINE1* methylation levels differed significantly (*p* = 0.009) between the three groups (ICSI, conventional insemination and controls). Furthermore, both the ART subgroups showed significant difference from the controls on post hoc analysis (Table 3).

### Oxygen tension

The embryos in the ART group were cultured either at atmospheric oxygen tension (20%) or physiological oxygen tension (5%). Both oxygen tension ART groups differ significantly in CCGG methylation levels from controls (*p* < 0.01). ANOVA indicated a significant difference between the groups (*p* = 0.0009) (Table 2). However, samples from individuals cultured in 20% oxygen did not differ from those cultured in 5% oxygen.

The *LINE1* methylation levels between the three groups (5% oxygen tension, 20% oxygen tension, and controls) also differed significantly (*p* = 0.0135). However, in this instance, the control group showed a significant difference with the 20% oxygen group (*p* < 0.05) but not the physiologic oxygen group (Table 3).

### Type of embryo transfer

ART samples were stratified into fresh and frozen subgroups.

Overall, these subgroups and controls have a significant difference in CCGG methylation levels (*p* < 0.0001). Post hoc analysis showed significant methylation differences in both the ART groups compared to controls (*p* < 0.05) (Table 2).

Similarly, we observed significant differences (*p* = 0.0137) in *LINE1* methylation levels between the three groups (fresh, frozen, and controls) (Table 3). Furthermore, the fresh group differed significantly from controls (*p* < 0.05) but the frozen group did not.

### Embryo transfer day

We analyzed global methylation levels based on the stage at which the embryo was transferred back to the uterus; cleavage stage (day 3) or blastocyst stage (day 5).

Again, these subgroups and controls were significantly different in CCGG methylation levels (Table 2; *p* = 0.0007), but these ART subgroups did not differ significantly from each other (data not shown). However, each of these subgroups had significantly different methylation levels compared to controls (*p* < 0.05) (Table 2).

We found a significant difference in *LINE1* methylation between controls, day 3 and day 5 embryo transfers (*p* = 0.0137). On applying Tukey’s post hoc test, both the embryo transfer subgroups showed significant methylation difference from the controls (*p* < 0.05) (Table 3).

### Sex of newborns

Although there was no significant difference in the sex ratio of ART vs control offspring, it is appropriate to ask whether the methylation differences observed between the ART and control groups are restricted to one sex because sex differences in both global and site-specific methylation levels have been observed [24, 29–34]. Stratifying the samples according to sex demonstrates that significant methylation differences are found in both the female (*p* = 0.0226) and the male (*p* = 0.0030) subgroups for CCGG sites (Additional file 1). However, *LINE1* elements showed significant difference in methylation levels only for the male (*p* = 0.0015) subgroup. Furthermore, female ART placentas did not show significant differences for any of the modifiable factors (data not shown). On the other hand, both male ART groups were significantly different (*p* < 0.05) from controls based on the mode of egg fertilization and embryo transfer day (Table 4). In addition, the 20% oxygen tension group (*p* < 0.01) and placentas from fresh embryo transfer (*p* < 0.05) showed significantly different *LINE1* methylation levels compared to the controls. Such differences were not evident for physiologic oxygen tension or frozen embryo transferred groups (Table 4).

**Table 4:** *LINE1* methylation pyrosequencing assay for males

Criteria for comparison	Groups (*n*)	Global methylation fraction (mean ± SD)	*P* value	
			ANOVA	Tukey’s post hoc test (vs controls)
	Controls (33)	0.5091 ± 0.0463	–	–
Mode of egg fertilization	ICSI (19)	0.4739 ± 0.0339	*0.0057*	**<** *0.05*
	Non ICSI (37)	0.4809 ± 0.0434		**<** *0.05*
Oxygen tension	20%(31)	0.4738 ± 0.0404	*0.0044*	**<** *0.01*
	5%(25)	0.4843 ± 0.0399		ns
Type of embryo transfer	Fresh (45)	0.4751 ± 0.0386	*0.0033*	**<** *0.01*
	Frozen (11)	0.4924 ± 0.0457		ns
Embryo transfer day	Day 3 (28)	0.4779 ± 0.0435	*0.0067*	**<** *0.05*
	Day 5 (28)	0.4791 ± 0.0375		**<** *0.05*

*ANOVA* analysis of variance; *SD* standard deviation; *ICSI* intracytoplasmic sperm injection; *ns* non significant

Values in italics denote significance

### Sex differences

We observed methylation differences in *LINE1* elements between male and female controls (*p* = 0.0494). However, ART placentas did not show (*p* = 0.4727) any sex-related methylation differences (Table 5). We obtained similar results in the validation cohort; sex-related differences were observed in controls (*p* = 0.0078) but not in ART placentas (*p* = 0.2149). The lack of a sex-difference among the ART population was confirmed by examining placentas from opposite sex twins (Table 5). The sex-specific methylation differences observed in controls do not appear among ART opposite sex twins (Table 5).

**Table 5:** Sex differences for *LINE1* methylation pyrosequencing assay

Cohort	Group	Males		Females		*P* values^a^
		Number of placentas	Global methylation fraction (mean ± SD)	Number of placentas	Global methylation fraction (mean ± SD)	
Original cohort	Controls	33	0.5091 ± 0.0463	32	0.4902 ± 0.0448	*0.0494*
	ART	56	0.4785 ± 0.0402	70	0.4791 ± 0.0503	0.4727
Validation cohort	Controls	29	0.4266 ± 0.0401	28	0.3936 ± 0.0583	*0.0078*
	ART	26	0.4227 ± 0.0382	29	0.4386 ± 0.09538	0.2149
Opposite sex twins	ART	39	0.4076 ± 0.0294	39	0.4098 ± 0.0320	0.3199

*ART* assisted reproductive technology; *SD* standard deviation

^a^Unpaired one-tailed *t* test

Values in italics denote significance [59]

## Discussion

We have observed that placentas from ART conceptions differ from control placentas in DNA methylation levels, whether examined CpG site-specifically [1, 35] or at hundreds-of-thousands-to millions of sites, simultaneously (Fig. 1; Tables 2 and 3). Notably, both hypomethylation and hypermethylation of ART samples are observed in both site-specific assays [1, 35] and at multiple-interrogated sites in different sequence contexts (Fig. 1; Tables 2 and 3). Given that the range of methylation values observed in ART and control populations overlaps to a great degree (Fig. 1), one may legitimately ask what is the source of the statistically significant differences observed. Our hypothesis is that the differences are due to a relatively small fraction of individuals in the ART group who have highly disrupted, so-called “outlier”, DNA methylation levels at a significant fraction of interrogated CpG sites [35]. We have observed that such individuals are more prevalent in “low-birth-weight” ART children [35] and multiple individuals who have hypermethylation at CCGG sites, compared with the most extreme controls, can be seen in Fig. 1a. An excess of such outliers is less obvious for hypomethylation of *LINE1* elements (Fig. 1b) but there are, in fact, many more ART individuals with *LINE1* methylation values less than 0.45 than control individuals.

Assisted reproductive technologies have been linked to epigenetic disorders and adverse neonatal outcomes [36, 37]. These anomalies may be the effect of parental factors (infertility or advanced age) [38], clinical and laboratory procedures used in ART or both parental factors and ART. We have observed that the site-specific DNA methylation differences found in autologous in vitro fertilized groups are also observed in children conceived with the aid of donor oocytes [10]. This observation suggests a role for ART procedures in causing these discrepancies but does not eliminate a role for parental factors. In addition to infertility, the most distinguishing characteristic of parents of ART children is older age. The present study clearly demonstrates that the global methylation differences between ART and control population are present even among the offspring of younger parents (≤35 years). This suggests that one or more of the interventions involved in ART could be responsible for these epigenetic differences, potentially leading to adverse outcomes in ART-conceived children.

The clinically modifiable ART interventions, such as superovulation, in vitro fertilization (conventional or ICSI), embryo culture (in 5 or 20% O_2_), and embryo transfer (cleavage or blastocyst stage), take place during the preimplantation embryonic stage during a time of extensive epigenetic reprogramming. We hypothesized that some or all of these interventions might influence the establishment and maintenance of epigenetic marks, leading to abnormal DNA methylation levels.

While both CCGG site methylation and *LINE1* methylation differed between ART and control groups, only *LINE1* methylation discriminated between different ART interventions. The reasons for this difference in discriminatory power between surrogate global measures of DNA methylation are unclear. CCGG sites are enriched in hypomethylated CpG islands [39, 40], which are found near the promoters of approximately half of all genes, while *LINE1* elements are dispersed in repeated sequences that are generally hypermethylated [40]. ART is associated with an increase in methylation in the usually hypomethylated CCGG site (Table 2) and a decrease in methylation in the usually hypermethylated *LINE1* sites (Table 3). In this regard, *LINE1* elements are normally protected from the wave of genome-wide demethylation that occurs during preimplantation development [41]. The maintenance of *LINE1* elements in the methylated state in preimplantation embryos has been suggested to suppress retrotransposition [23]. The fact that ART placentas, overall, and each ART intervention, individually, leads to lower levels of *LINE1* methylation (Table 3) suggest the possibility that ART interventions may result in hypomethylation of some *LINE1* elements and a corresponding level of new *LINE1* retrotranspositions in the genome.

CCGG site methylation did not reveal any differences among the different interventions, as both ART groups within an intervention differ significantly from the controls, without significant differences between the interventions being compared. However, *LINE1* methylation did show differences between the ART groups in the oxygen tension of embryo culture and embryo transfer type.

It has been argued earlier that embryo culture at physiologic oxygen tension (5%) has a better success rate [42]. Studies in mice have shown that 20% O_2_ delays oocyte maturation, thereby impairing the development of oocytes [43] and adversely affecting the developmental potential of blastocysts [44]. Low concentrations of reactive oxygen species have been observed in mouse embryos cultured in 5% O_2_ compared to those from 20% O_2_ [45]. In human IVF studies, physiologic oxygen concentration has been found to improve the blastulation rate [46], blastocyst yield and embryo quality [47], and increase live births [42]. Cochrane Database Review [48] also supports the view that physiologic oxygen tension has a better success rate. Our data (Table 3) also suggest a detrimental effect of 20% oxygen over 5% oxygen tension on the epigenome, in comparison with controls, as assayed by global *LINE1* methylation.

Superovulation is an integral part of IVF procedure wherein women undergoing IVF are administered exogenous gonadotropins to stimulate the production of multiple follicles. This results in serum estradiol levels 10 times greater in a fresh IVF cycles than observed during a natural cycle [49]. This hormonally imbalanced uterine environment has been shown to affect embryo implantation [50] and fetal growth [51] in mice. Frozen embryo transfers (FET) have been found to overcome these adverse effects of supraphysiologic hormonal levels and improve endometrial receptivity [52]. A recent meta-analysis of 13 cohort studies concluded that FET reduces the risks of preterm birth and low birth weight [53]. At the expression level, frozen embryos showed more consistent gene expression over morphologically matched fresh embryos [54]. Our data suggests that global methylation levels of frozen transfers are consistent with the control group but these are significantly variable for the fresh transfers.

The other interventions studied were the effects of ICSI vs conventional IVF and day 3 vs day 5 of embryo transfer. Our data are inconclusive on whether either modification of either of these practices is less disruptive to the epigenome. Others have found that ICSI is associated with more epigenetic alterations compared to conventional IVF [55], but there is no clear indication as to whether day 3 or day 5 embryo transfer is better. A recent Cochrane Database Review found moderately high clinical pregnancy and live birth rates in blastocyst fresh transfers, but the data was inconclusive in regards to cumulative live birth and pregnancy rates [56].

Our sex-stratified analysis suggests that sex of the newborns could have a major role in influencing the observed methylation differences between ART and control placentas. In our study, CCGG methylation showed significant differences for both male and female subgroups, but the *LINE1* methylation data showed differences in male subgroups only. On further analysis based on the modifiable factors, the males showed similar results as observed in the combined analysis (males and females). On the other hand, we did not see any differences in the female group. This led us to investigate the methylation difference between the sexes. We found significant methylation difference between males and females in controls, but this difference is not present in the ART population. The absence of sex-influenced methylation differences in ART was validated in an additional cohort and in opposite sex twins conceived by ART.

There are reports showing that *LINE1* methylation is influenced by sex [24, 57]. *LINE1* hypomethylation in females was attributed to dietary differences and decreased circulatory folate levels due to menstruation in these studies. However, it is unclear why this sex difference is not evident in the ART children.

Males are more sensitive to maternal obesity induced inflammation [58], suggesting a greater impact of in utero environment on male fetuses. Such a sex bias in sensitivity to in utero environmental exposures could provide a possible explanation for the sex-specific effect of ART on expunging male/female epigenetic differences that exist in in vivo-conceived offspring. Further studies are needed to evaluate these sex specific differences in depth.

There are limitations to this study. Clearly, other factors could be affecting DNA methylation, including culture media, response to superovulation, maternal BMI as well as the many other unknown factors that might affect the epigenome. In addition, long-term clinical significance of these changes in global methylation must still be investigated. However, these findings strengthen previous data, from our group and others, that techniques utilized during ART lead to changes in DNA methylation.

## Conclusions

We conclude that two clinically modifiable factors (5 vs 20% oxygen tension of embryo culture and fresh vs frozen embryo transfer) are associated with global placental methylation differences. In both interventions, the subgroup associated with better clinical outcomes (5% O_2_ and frozen embryo transfer; [42–48, 52–54]) is also associated with global DNA methylation levels that are closer to those of children conceived in vivo. This suggests not only that DNA methylation may be responsible for (at least some) the adverse clinical outcomes associated with IVF, but that modification of our current practices may decrease the incidence of these adverse outcomes. Further investigations correlating changes in DNA methylation and adverse perinatal outcomes are necessary to establish the protocols that can minimize epigenetic changes following ART and reduce complications associated with IVF.

## Additional files

Additional file 1: Effect of parental age and sex of newborns. (DOCX 87 kb)
Additional file 2: Distribution of global methylation fractions based on modifiable factors: mode of egg fertilization by LUMA (A) and *LINE1* assay (B); oxygen tension by LUMA (C) and *LINE1* assay (D); type of embryo transfer by LUMA (E) and *LINE1* assay (F) and; embryo transfer day by LUMA (G) and *LINE1* assay (H). (PPTX 385 kb)
