# Supplementary material for: Global DNA methylation levels are altered by modifiable clinical manipulations in assisted reproductive technologies
Source: Clin Epigenetics. 2017 Feb 6;9:14. doi: 10.1186/s13148-017-0318-6 (PMC5295214; doi:10.1186/s13148-017-0318-6)
Supplement: Additional file 1: — Effect of parental age and sex of newborns. (DOCX 87 kb) [file 13148_2017_318_MOESM1_ESM.docx]

**Additional file 1: Effect of parental age and sex of newborns**

|  | ART | | Controls | |  |
| --- | --- | --- | --- | --- | --- |
| Groups | Number of samples | Global Methylation Fraction  (Mean±SD) | Number of samples | Global Methylation Fraction  (Mean±SD) | P value^a^ |
| LUMA |  |  |  |  |  |
| Maternal age <35 | 88 | 0.5176±0.0596 | 55 | 0.4793±0.0758 | **0.0010** |
| Maternal age≥35 | 94 | 0.5188±0.0736 | 22 | 0.4914±0.0659 | 0.1113 |
| Paternal age <35 | 72 | 0.5186±0.0651 | 37 | 0.4657±0.0759 | **0.0002** |
| Paternal age≥35 | 103 | 0.5160±0.0696 | 26 | 0.5004±0.0713 | 0.3102 |
| Females | 99 | 0.5183±0.0699 | 38 | 0.4879±0.0667 | **0.0226** |
| Males | 83 | 0.5182±0.0637 | 39 | 0.4777±0.0789 | **0.0030** |
| LINE-1 |  |  |  |  |  |
| Maternal age <35 | 64 | 0.4720±0.0338 | 49 | 0.5011±0.0495 | **0.0003** |
| Maternal age≥35 | 62 | 0.4860±0.0551 | 16 | 0.4956±0.0355 | 0.5084 |
| Paternal age <35 | 51 | 0.4722±0.0398 | 35 | 0.4982±0.0517 | **0.0100** |
| Paternal age≥35 | 70 | 0.4852±0.0504 | 21 | 0.4924±0.0399 | 0.5535 |
| Females | 70 | 0.4791±0.0503 | 32 | 0.4902±0.0449 | 0.2892 |
| Males | 56 | 0.4785±0.0402 | 33 | 0.5091±0.0463 | **0.0015** |

a unpaired two tailed t-test

Values in bold denote significance
