# Supplementary figures and images for: Global DNA methylation levels are altered by modifiable clinical manipulations in assisted reproductive technologies
Source: Clin Epigenetics. 2017 Feb 6;9:14. doi: 10.1186/s13148-017-0318-6 (PMC5295214; doi:10.1186/s13148-017-0318-6)

## Slide 1
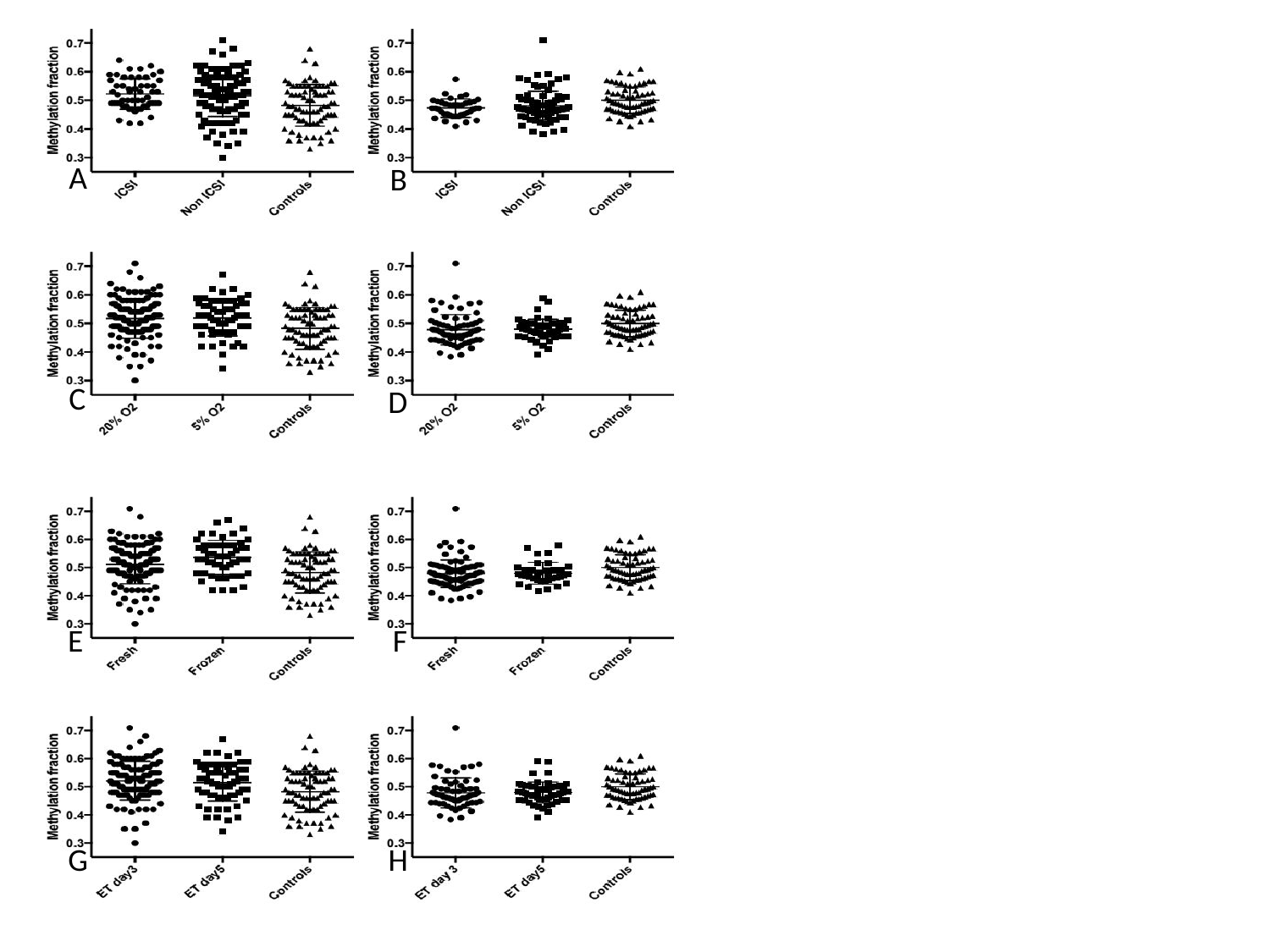

A
B
C
D
E
F
G
H

Supplement: Additional file 2: — Distribution of global methylation fractions based on modifiable factors: mode of egg fertilization by LUMA (A) and LINE1 assay (B); oxygen tension by LUMA (C) and LINE1 assay (D); type of embryo transfer by LUMA (E) and LINE1 assay (F) and; embryo transfer day by LUMA (G) and LINE1 assay (H). (PPTX 385 kb) [file 13148_2017_318_MOESM2_ESM.pptx]
